# Quantitative transcript analysis of the inducible expression system pSIP: comparison of the overexpression of *Lactobacillus *spp. β-galactosidases in *Lactobacillus plantarum*

**DOI:** 10.1186/1475-2859-10-46

**Published:** 2011-06-22

**Authors:** Tien-Thanh Nguyen, Thu-Ha Nguyen, Thomas Maischberger, Philipp Schmelzer, Geir Mathiesen, Vincent GH Eijsink, Dietmar Haltrich, Clemens K Peterbauer

**Affiliations:** 1 Food Biotechnology Lab, Department of Food Sciences and Technology, University of Natural Resources and Life Sciences Vienna, Austria; 2 Research Centre Applied Biocatalysis, Graz, Austria; 3 Department of Chemistry, Biotechnology and Food Science, Norwegian University of Life Sciences, Ås, Norway; 4 School of Biotechnology and Food Technology, Hanoi University of Science and Technology (HUST), Hanoi, Vietnam

## Abstract

**Background:**

Two sets of overlapping genes, *lacLMReu *and *lacLMAci*, encoding heterodimeric β-galactosidases from *Lactobacillus reuteri *and *Lactobacillus acidophilus*, respectively, have previously been cloned and expressed using the pSIP vector system and *Lactobacillus plantarum *WCSF1 as host. Despite the high similarity between these *lacLM *genes and the use of identical cloning and expression strategies, strains harboring *lacLMReu *produced about twenty-fold more β-galactosidase than strains containing *lacLMAci*.

**Results:**

In this study, the plasmid copy numbers (PCN) of expression vectors pEH9R (*lacLMReu*) and pEH9A (*lacLMAci*) as well as the transcription levels of both *lacLM *genes were compared using quantitative PCR methods. Analyses of parallel fermentations of *L. plantarum *harboring either pEH9R or pEH9A showed that the expression plasmids were present in similar copy numbers. However, transcript levels of *lacLM *from *L. reuteri *(pEH9R) were up to 18 times higher than those of *lacLM *from *L. acidophilus *(pEH9A). As a control, it was shown that the expression levels of regulatory genes involved in pheromone-induced promoter activation were similar in both strains.

**Conclusion:**

The use of identical expression strategies for highly similar genes led to very different mRNA levels. The data indicate that this difference is primarily caused by translational effects that are likely to affect both mRNA synthesis rates and mRNA stability. These translational effects thus seem to be a dominant determinant for the success of gene expression efforts in lactobacilli.

## Background

Lactic acid bacteria (LAB) are important micro-organisms in the food and beverages industry. Over the past few decades, LAB have been used not only as starter culture but also as producers of flavoring enzymes, antimicrobial peptides or metabolites that contribute to the flavor, texture and safety of food products [1-3]. Moreover, because of their food-grade status and probiotic characteristics, several LAB, especially lactobacilli, are considered as safe and effective cell factories for food-application purposes [2,3]. As a consequence, a variety of constitutive or inducible gene expression and protein targeting systems for LAB hosts have been developed, including sugar-inducible, thermo-inducible and pH-dependent expression systems [1,2,4].

Two well-known inducible expression systems for LAB exploit promoters from bacteriocin operons, the NIsin-Controlled Expression system (NICE) [5] and the pheromone-inducible system pSIP [6]. The NICE system exploits genes and promoters involved in the production of the antimicrobial peptide (lantibiotic) nisin in *Lactococcus lactis *and the inducing substance is nisin itself [5]. Similarly, the pSIP systems were developed based on promoters and regulatory genes involved in the production of the class II bacteriocins sakacin A [7] and sakacin P [8,9] in *Lactobacillus sakei*. In these LAB, bacteriocin production is regulated by a three-component system, consisting of a secreted peptide pheromone (IP) which interacts specifically with a cognate membrane-embedded histidine protein kinase (HPK). A response regulator (RR) encoded in the same operon as IP and HPK is activated by the HPK, leading to induction of all the promoters of the bacteriocin operons [8]. The pSIP systems have been used to over-produce several enzymes such as β-glucuronidase and aminopeptidase N in several *Lactobacillus *hosts [6,10,11].

β-Galactosidases (lactase, EC 3.2.1.23) are known as important enzymes in the dairy industry [12-14]. The ability of β-galactosidases to convert lactose into galactose and glucose is used to prevent the crystallization of lactose, to improve sweetness, to increase the solubility of milk products, and to produce lactose-free food products [15]. Another beneficial ability of β-galactosidases is the trans-galactosylation reaction which co-occurs during lactose hydrolysis [12] and results in the formation of galacto-oligosaccharides (GOS). Similar to fructo-oligosaccharides (FOS), GOS possess prebiotic properties [13,14,16,17].

Many β-galactosidases of lactobacilli, including the enzymes from *L. reuteri *and *L. acidophilus*, consist of two subunits, one large and one small, which are encoded by two overlapping genes, *lacL *and *lacM*, respectively [13]. In a previous study, we have overexpressed the β-galactosidases from *L. reuteri *L103 and *L. acidophilus *R22 by cloning the *lacLM *genes into pSIP vectors [10]. Two of the resulting expression vectors, pEH9R and pEH9A, are based on pSIP409 and contain *lacLMReu *from *L. reuteri *L103 and *lacLMAci *from *L. acidophilus *R22, respectively [10]. The *lacLM *genes are under the control of the strong pheromone-inducible promoter P_sppQ _[6,11,18], to which they are translationally fused, and over-expression of these β-galactosidases in the well-studied food-grade strain *Lactobacillus plantarum *WCFS1 was successful. However, even though the amino acid sequences of these β-galactosidases are highly similar, both SDS-PAGE analyses of cell extracts and activity measurements showed that the two enzymes had very different production levels under identical conditions, with *lacLMReu *being expressed about twenty times more efficiently than *lacLMAci *[10].

The observed expression levels are the end product of transcription, translation and post-translational processes, which all may be influenced by a large number of factors, including the gene dose, which is determined by the plasmid copy number (PCN), and messenger-RNA (mRNA) levels. In the present study we have used RT-qPCR to verify whether the different expression efficiencies of *lacLMAci *and *lacLMReu *correlate with differences in mRNA levels. Furthermore, we used RT-PCR to determine the plasmid copy numbers of pEH9A and pEH9R. Since identical cloning strategies had been used for highly similar genes, substantial differences were not a priori expected. Interestingly, however, large differences in mRNA levels were found.

## Materials and methods

### Bacterial strains and media

*Lactobacillus plantarum *WCFS1 [19], harboring pEH9R or pEH9A containing the overlapping genes (*lacLM*) encoding β-galactosidase of *Lactobacillus reuteri *L103 and *Lactobacillus acidophilus *R22, were maintained in MRS (Merck, Darmstadt, Germany) containing 5 μg/ml erythromycin and 15% glycerol at -70°C.

### Fermentations

Strains were activated from frozen stock in 5 ml of MRS with 5 μg/ml erythromycin at 37°C for 16-18 h. These cultures were used to inoculate 400 ml MRS medium (40 g/l glucose, 5 μg/ml erythromycin). Cultivations were done in an HT-Multifors system (Infors HT, Switzerland) with pH control at pH 6.5, at 37°C. Sodium hydroxide was used for maintaining the pH. A low agitation speed (200 rpm) was set to ensure the homogeneity of medium and other parameters and to ensure continuous contact between bacterial cells and nutrient. Induction of gene expression was achieved by adding a 19-amino acid synthetic peptide pheromone, IP673, with a sequence identical to the sequence of the pheromone as originally isolated from *Lactobacillus sakei *LTH673 [20].

Growth of bacteria was monitored via the optical density at 600 nm (OD_600_). After six hours, when OD_600 _had reached around 3.0, IP673 was added to a final concentration of 80 ng/ml to induce *lacLM *gene transcription. Samples were collected at intervals for OD_600 _measurements, enzyme assays, and DNA and RNA isolation.

For β-galactosidase measurements, cells from 1 ml of fermentation broth were pelleted by centrifugation at 13200 rpm for 3 min. Cells were re-suspended in buffer P [16], then disrupted by sonication (4 × 1 min at 100% power, interrupted by 1 min breaks and constant cooling on ice, using a Bandelin Sonopuls HD60, Berlin, Germany). Subsequently, cell debris was removed by centrifugation at 13200 rpm for 10 min at 4°C. The obtained crude extract was used for measuring β-galactosidase activity as well as protein concentration. For DNA or RNA isolation, cells were pelleted as described, shock-frozen by liquid nitrogen and stored at -80°C until further use.

### Enzyme assays

β-Galactosidase activity was determined using *o*-nitrophenyl-β-D-galactopyranoside (*o*NPG) as previously described [13]. Protein concentration was determined using the method of Bradford [21] with bovine serum albumin as standard.

### Bacterial DNA isolation and purification for PCN estimation

A sample (1 ml) of the culture at an OD_600 _of 10 was used for DNA isolation. For cells harvested at lower values of OD_600_, correspondingly higher culture volumes were collected (e.g., 2 ml of a culture with an OD_600 _of 5). DNA was isolated and purified using the phenol-chloroform extraction method as described in literature [22]. The purified bacterial DNA was stored at -20°C until use.

### RNA isolation and purification

Total RNA was isolated using the peqGOLD Bacterial kit (Peqlab, Biotechnologie GmbH, Germany) according to the supplier's instructions without DNA on-column digestion. The concentration of total RNA was determined spectrophotometrically at 260 nm (A_260_) (Beckman DU80). RNA integrity was examined by denaturing agarose gel electrophoresis (2% agarose, 2.2 M formaldehyde). DNA contaminations in total RNA samples were completely removed by digestion with 1 U/μl of DNAse (PeqLab) in a total volume of 20 μl using DNAse reaction buffer as recommended [23]. After 10 min at 37°C, 30 mM EDTA solution was added to a final concentration of 3 mM. The mixture was heated at 70°C for 15 min to inactivate DNAse and stored at -70°C. The absence of residual DNA contamination was confirmed by normal PCR with 16S primer pair (not shown).

### Reverse transcription and real time quantitative PCR

#### Reverse transcription

RNA was reverse-transcribed using the First Strand cDNA Synthesis kit (Fermentas, St. Leon-Roth, Germany) with random hexamer priming and 100 units of reverse transcriptase (RevertAidTM M-MuLV Reverse Transcriptase, Fermentas). The resulting cDNAs were stored at - 20°C. For control samples, dimethylpyrocarbonate (DEPC) treated water was used to replace reverse transcriptase.

#### Oligonucleotide primers

Oligonucleotide primers used in this study are listed in Table 1. For plasmid copy number estimation, the erythromycin resistance gene *ermB *and *16SrRNA *were chosen as representatives for plasmid DNA and genomic DNA, respectively. From the sequences of these genes, two primer pairs called Ery^R ^and 16S were designed (Table 1).

**Table 1:** Oligonucleotide primers used in this study

Primer	**Sequence 5' - 3'**^**a**^	**T**_**a**_^**b **^**(°C)**	**Concentration**^**c **^**(nM)**	Product size (bp)	Tm product (°C)
16S_f	TGATCCTGGCTCAGGACGAA	60	250	81	81

16S_r	TGCAAGCACCAATCAATACCA		250		

Ery^R^_f	CCGTGCGTCTGACATCTAT	60	250	108	79

Ery^R^_r	TGCTGAATCGAGACTTGAGTG		250		

LacReu_f	CCA GAT TCC GTG GTA TTA CCT TTG TG	60	250	154	80

LacReu_r	TAC TACT ACG TCA CGC CAT TGA GGA AC		500		

LacAci_f	TCTAGTTCACTACGAAGGTGTCG	60	500	154	76.5

LacAci_r	GTCATGCATGTATTCACACTCC		500		

SppKR_f	CAAGCCGTTCAAGAAACCGAT	60	250	144	78.5

SppKR_r	AGCGCCTTTCGTTGAATAGCC		500		

11n15_f	GATGACCCGGAAATTTTTCGCGTCAATCAATTGCCAGC				

11n15_r	GACGCGAAAAATTTCCGGGTCATC				

**^a ^**exchanged codons in 11n15_f and 11n15_r are underlined

**^b ^**optimized annealing temperature as described in the Materials Section

^**c**^optimized concentration as described in the Materials Section

For relative mRNA quantification of the three genes *lacLMReu*, *lacLMAci *and *sppKR*, 3 primer pairs called LacReu, LacAci and SppKR, respectively, were designed. The two former primer pairs were designed so that their characteristics as well as the length of amplicons were similar. The chromosomal*16SrRNA *gene was used as reference gene.

#### RT-PCR reaction using SYBR Green I dye

The thermal cycling system iCycler and myIQ single Color Real-Time PCR Detection system (BioRad, Hercules, CA) were used for qPCR amplification and detection. The qPCR reactions (25 μl total volume) were prepared in duplicates in 96-wells plates (BioRad) that were sealed with optical adhesive covers (Microseal 'B' film, BioRad). Each reaction included an optimized concentration for each of forward and reverse primers (see Table 1), 12.5 μl of Perfecta SYBR Green Super mix of IQ (Quanta Biosciences), and 2.5 μl of DNA template. Negative controls (no template control), prepared by replacing the DNA template with DEPC water, were included in each run to confirm the absence of DNA contaminations in the reagents. Before setting up the experiments described in the Results section, primer concentrations, annealing temperatures and DNA template concentrations were optimized according to procedures and criteria described in [23], and the final optimized reaction parameters are shown in Table 1.

The qPCR reactions were conducted as follows: initial denaturation at 95°C for 3 min followed by 50 cycles of 20 s at 95°C, 20 s at 60°C, and 72°C for 10 s. The fluorescence signal was collected at the end of each extension step at 72°C. Afterwards, the temperature was increased from 55°C to 95°C at a rate of 0.2°C/s to establish the melting curve.

The threshold cycle values (C_t_) were automatically determined by the software Biorad MyIQ optical system Version 2.0.

#### Calculation of the PCN value

Based on the PCN definition, which is the number of copies of a plasmid present per chromosome in bacteria [24,25], the PCN can be calculated by the following equation [26]:  (1), here, *E_c_, C_tc _*and *E_p_, C_tp _*are the amplification efficiency and the threshold cycle value of the amplicon representing chromosome and plasmid, respectively. The equivalence between the amplification efficiency (E) of plasmid and chromosome amplicons was confirmed in validation experiments as recommended [27]. In addition, to compare the PCN between two recombinants, the relative PCN values were calculated using the comparative C_t _method (ΔΔC_t_), in the following equation:  (2), where ΔΔC_t _= ΔC_t _of the sample corresponding to pEH9R - ΔC_t _of sample corresponding to pEH9A, and ΔC_t _= average C_t _value of target (for erythromycin resistance gene) - average C_t _value of reference gene.

#### Calculation of the expression ratio

The relative expression level between the two genes (e.g. A and B) was also estimated as described in equation (2), where ΔΔC_t _= ΔC_t _corresponding to gene A - ΔC_t _corresponding to gene B and ΔC_t _= average C_t _of target genes (A or B) - average C_t _of reference gene (*16S rRNA*).

The relative expression level of each gene of interest compared to the time point before induction (here after 6 h of cultivation) was estimated accordingly, but ΔΔC_t _= ΔC_t _of genes of interest at different time points - ΔC_t _of genes of interest after 6 h of cultivation. In the present work, the genes of interest were *lacLMReu*, *lacLMaci *and *sppKR*.

#### Codon usage and mRNA secondary structure analysis

The codon usage of the *lacLM *genes was compared to those of *L. plantarum *WCFS1 using the Graphical Codon Usage Analyzer (http://gcua.schoedl.de/index.html). The codon usage table of *L. plantarum *WCFS1 is estimated based on 3057 CDS's (934462 codons) (http://www.kazusa.or.jp/codon/cgi-bin/showcodon.cgi?species=220668). mRNA secondary structure for both genes was analyzed using mfold (http://mobyle.pasteur.fr/cgi-bin/portal.py?form=mfold) from the transcription start point (65 nt upstream of ATG) to 150 nt (50 codons).

### Expression of a mutated variant of the *L. acidophilus lacLM *gene

To exchange the triplets 11 and 15 of the *L. acidophilus lacLM*-coding region the overlapping primers 11n15_f and 11n15_r (Table 1) were used. Site-directed mutagenesis PCR was performed in 25-μl reaction volume with Phusion High Fidelity DNA Polymerase (Finnzymes, Espoo, Finland) using pEH9A as the template (annealing temperature of 52°C). The residual template after amplification was digested by 1 μl *Dpn*I (20 U) (Fermentas) for 4 h at 37°C. The reaction products were purified using the Wizard^® ^SV Gel PCR Clean-Up system (Promega, Madison, WI) and transformed into *E. coli *NEB5α. Several randomly picked transformants appearing after 24 h incubation at 37°C were checked by sequencing of the isolated plasmids. A plasmid with verified mutations at triplets 11 and 15 and no additional changes was selected and named pEH9A1. To exclude possible undesired mutations in the (non-sequenced) plasmid backbone, the 3.3-kb *Spe*I-*Eco*RI fragment from pEH9A1 was ligated into a 5.2-kb *Spe*I-*Eco*RI-fragment from pEH9A, resulting in the plasmid pEH9A2. This plasmid was electroporated into competent cells of *L. plantarum *WCFS1. *L. plantarum *WCFS1 harboring pEH9R, pEH9A2 and pEH9A were cultivated and induced in parallel in a Multifors fermenter as described above. Harvested cells were disrupted using 1 gram glass beads in a Precellys 24 bead mill (Peqlab). The cell-free extracts were obtained after a centrifugation step at 13200 rpm/10 min at 4°C and used for enzyme assays and protein analysis.

Experiments in this manuscript were conducted in accordance with the Austrian Gentechnikgesetz (GTG). No experiments requiring approval by an ethics commission are described in the manuscript.

## Results

### Fermentations

**Figure 1:**
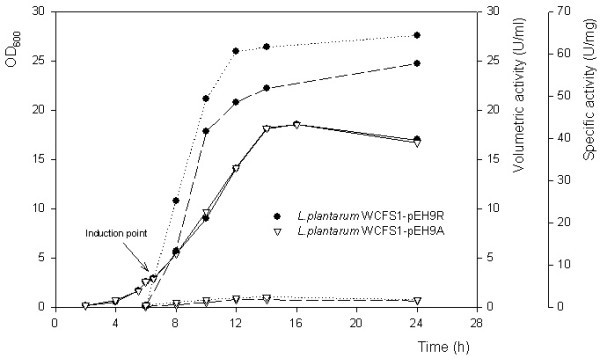
**Time course for growth of *L. plantarum *WCFS1 carrying pEH9R or pEH9A**. Cultivations were carried out with pH control at pH 6.5 in 400-ml laboratory fermentors at 37°C using MRS medium (40 g/l glucose). The graph shows OD_600 _(solid lines), β-galactosidase activity (units per milliliter of fermentation broth) (dashed lines) and specific activity (units per milligram protein) (dotted lines). Cultures were induced with 80 ng/ml of pheromone after six hours of growth, i.e. at an OD_600 _of approximately 3.0.

*L. plantarum *WCFS1 harboring either pEH9R or pEH9A (carrying *lacLMReu *and *lacLMAci*, respectively, under the control of the P_sppQ _promoter) was cultivated in a pH-controlled fermentor using conditions that had previously been determined to result in high enzyme yields (unpublished data). Gene expression was induced by adding the peptide pheromone IP-673 6 h after the start of the fermentation (at an OD_600 _of approximately 3.0). Results presented in Figure 1 show that growth of the two strains was nearly identical over the entire fermentation. In contrast, β-galactosidase yields (in terms of both units per volume of fermentation broth and units per mg protein) were considerably different. *L. plantarum *WCFS1 carrying pEH9A showed a maximum activity around 0.8 U/ml and 2.5 U/mg, whereas with pEH9R maximum activities reached about 22 U/ml and 62 U/mg (Figure 1). SDS-PAGE experiments confirmed that these differences are correlated with large differences in protein production levels, as was observed in earlier work with these constructs [10]. Previous studies have shown that the purified β-galactosidases from *L. reuteri *and *L. acidophilus *have similar specific activities [13,16].

### Plasmid copy numbers

Plasmid copy numbers (PCN) in cells harboring pEH9R or pEH9A were compared after 6 h (point of induction), 8 h, 12 h and 24 h of cultivation. PCN ratios for pEH9R versus pEH9A varied between 1.39 and 0.79, i.e. close to one, in all cases (Table 2). Thus, both plasmids had similar copy numbers throughout the fermentation. The PCN for pEH9R in *L. plantarum *WCFS1 was determined to be approximately three to four.

**Table 2:** β-Galactosidase activity and transcript levels

Time (h)	Time after induction (h)	pEH9R			PCN ratio pEH9R/pEH9A	pEH9A		
				
		**Activity **^**a**^	lacLM expression level	sppKR expression level		**Activity **^**a**^	lacLM expression level	sppKR expression level
6 ^b^	0	1	1.00 ± 0.07	1.00 ± 0.16	1.39 ± 0.25	1	1.00 ± 0.13	1.00 ± 0.21

8	2	56.1	59.9 ± 15.6	1.88 ± 0.11	0.81 ± 0.07	2.42	17.8 ± 4.3	2.49 ± 0.04

12	6	135	55.4 ± 13.5	1.67 ± 0.39	0.79 ± 0.01	4.48	11.9 ± 1.9	2.00 ± 0.20

24	18	144	16.4 ± 2.6	-	1.34 ± 0.06	3.56	0.46 ± 0.12	-

Activity levels and transcript levels of *lacLM *and *sppKR *in strains harboring pEH9R or pEH9A are related to the respective values at the induction point (6 h into the cultivation)

The plasmid copy number (PCN) ratio is the value for the pEH9R-harboring strain divided by the value for the pEH9A-harboring strain

^a ^Specific activity (U/mg protein)

^b ^Just before induction

### Transcription level of *lacLM *genes

To study mRNA levels and the effect of induction on the expression of *lacLM *genes, the relative expression of *lacLMReu *and *lacLMAci *at several time points (2, 6 and 18 h after induction, i.e. 8, 12 and 24 h after start of the fermentation) was compared to the expression of these genes just before induction (6 h after start of the fermentation). Table 2 shows strongly increased expression of the *lacLM *genes 2 and 6 h after induction. Messenger-RNA levels for *lacLMReu *showed an approximately 60-fold increase after 8 h (2 h after induction), whereas mRNA levels for *lacLMAci *were increased about 18-fold at the same time point. Subsequently, mRNA levels decreased and they did so faster for *lacLMAci *than for *lacLMReu*. After 24 h of cultivation (18 h after induction) mRNA levels for *lacLMReu *were still considerably elevated, whereas mRNA levels for *lacLMAci *were lower than before induction (Table 2).

### Transcription levels of *sppKR*

Addition of peptide pheromone to the growth medium will induce the expression of *sppK *and *sppR*, and this autoinduction loop will increase the expression of *lacLM*. Although not likely, the strength of the expression of *sppKR *may vary between the two plasmids. Therefore, we analyzed mRNA levels for *sppKR *in the two strains harboring pEH9R or pEH9A, before and after induction (Table 2). Expression of *sppKR *indeed increased after induction, albeit by not more than approximately a factor two (Table 2). The ratio between the *sppKR *transcript levels in the strains harboring pEH9R or pEH9A was close to 1 at all tested time points, showing that the expression levels of *sppKR *were essentially identical in both strains. For both fermentations (pEH9R and pEH9A), the transcript level of *sppKR *was compared to that of the reporter genes, *lacLM*. Before induction, the mRNA level of *sppKR *was higher than the level of *lacLM *mRNA (approximately five-fold and 14-fold for pEH9R and pEH9A, respectively). After induction these ratios decreased to about 0.15 for pEH9R and 2.0 - 2.4 for pEH9A (Table 3), reflecting the much higher mRNA levels for *lacLMReu *after induction.

**Table 3:** Ratio of expression levels of *sppKR *versus *lacLM *in *L. plantarum *WCFS1 carrying pEH9R and *L. plantarum *WCFS1 carrying pEH9A

Time (h)	Time after induction (h)	pEH9R	pEH9A
6^a^	0	4.87 ± 0.78	14.32 ± 2.96

8	2	0.15 ± 0.01	2.01 ± 0.03

12	6	0.15 ± 0.03	2.40 ± 0.23

Values were obtained by dividing the average transcript number of *sppKR *with the average transcript number of *lacLM *as described in Material and Methods

^a ^Just before induction

### Codon usage analysis

**Figure 2:**
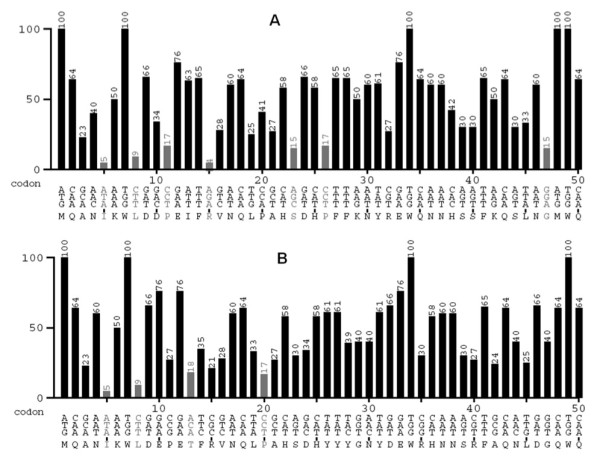
**Codon usage analysis of the 50 first codons in *lacLM *of *L. acidophilus *(A) and *L. reuteri *(B)**. The vertical axis indicates the codon usage frequency (%) of triplet codons in *L. plantarum *WCFS1 (from http://www.kazusa.or.jp/codon/cgi-bin/showcodon.cgi?species=220668). Codons with frequencies below 20% are considered rare and their frequencies appear in grey color.

The mean difference of codon usage in the *lacLM *genes from *L. reuteri *and *L. acidophilus *compared to the codon usage of *L. plantarum *WCFS1 was 16.48% and 23.45% (for *lacL*) and 18.22% and 25.75% (for *lacM*), respectively. The total numbers of "rare codons" (i.e., codons used in less than 20% of the cases) and "very rare codons" (less than 10%) are approximately equal in the *lacLM *genes from *L. reuteri *and *L. acidophilus*, but the latter shows a larger number of rare codons in the first 50 triplets of the *L. acidophilus *gene (seven, vs. four in the *L. reuteri *gene; see Figure 2).

### Expression of a mutated *L. acidophilus lacLM *gene

In order to investigate the influence of the observed differences in codon usage on transcription and possibly translation we constructed a mutated variant of the *L. acidophilus lacLM *gene. Triplets in codon 11 (CCT, encoding proline) and 15 (AGA, encoding arginine) (Figure 2A), which are considered very rare in *L. plantarum *WCFS1, were replaced with the corresponding codons from the *L. reuteri *gene, which are more common in *L. plantarum *WCFS1 (CCG and CGC, respectively; Figure 2B). The mutated gene (on the vector pEH9A2) was expressed in *L. plantarum *WCFS1 under the same conditions as described for pEH9A and pEH9R, using bacteria harboring these two plasmids as control. Resulting β-galactosidase activities in the cultures harboring pEH9A2 were indeed higher than in those harboring pEH9A, but only by roughly 20 - 40% (Table 4).

**Table 4:** β-Galactosidase activity of *L. plantarum *WCFS1 harboring pEH9R, pEH9A and pEH9A2^a^

Volumetric activity (kU/L)				
Time (h)	WCFSI + pEH9A2	WCFS1 + pEH9A	WCFS1 + pEH9R	Ratio (pEH9A2/pEH9A)

6	0.23 ± 0.00	0.17 ± 0.00	2.45 ± 0.10	1.43

8	1.34 ± 0.05	1.09 ± 0.08	12.0 ± 0.4	1.23

12	3.00 ± 0.12	2.40 ± 0.19	76.3 ± 4.1	1.25

24	3.17 ± 0.15	2.69 ± 0.31	101 ± 6	1.19

				

**Specific activity (U/mg)**				

Time (h)	WCFSI + pEH9A2	WCFS1 + pEH9A	WCFS1 + pEH9R	Ratio (pEH9A2/pEH9A)

6	1.15 ± 0.04	0.85 ± 0.08	16.1 ± 0.1	1.36

8	2.83 ± 0.08	2.60 ± 0.07	39.0 ± 3.4	1.09

12	3.18 ± 0.10	2.67 ± 0.13	95.3 ± 0.2	1.19

24	3.31 ± 0.41	2.85 ± 0.33	97.1 ± 5.7	1.16

^a ^The method used for cell disruption used in this study differed from the method used to produce Fig. 1 (see Materials and Methods). This explains why the absolute enzyme activity values vary between the two experiments.

## Discussion

We have previously shown that lactobacillal *lacLM *genes can be overexpressed in *Lactobacillus plantarum *WCFS1 using the inducible pSIP expression system [10]. In this previous work we observed remarkably large differences in expression levels of β-galactosidases from different *Lactobacillus *strains. In the present study, we have used optimized conditions (unpublished observations) in parallel fermentations of *L. plantarum *WCFS1 expressing different *lacLM *genes. Under conditions of pH control (pH 6.5) and high sugar content (see **Materials and Methods **section), the highest β-galactosidase activities were observed in the late exponential phase, where expression of *lacLMReu *yielded 65 U/mg protein compared to only 2.5 U/mg obtained with *lacLMAci*.

Plasmid copy numbers may have significant effects on the synthesis of recombinant proteins encoded by a plasmid-borne gene [25]. Plasmids pEH9R and pEH9A were both constructed using an identical pSIP409 backbone containing the 256rep replication determinant derived from the *L. plantarum *NC7 plasmid p256 [28]. Copy numbers of vectors with this origin of replication are rather low, and were determined to be approximately three in *L. sakei *Lb790 and six in *L. plantarum *NC8, using slot-blot hybridization [11,28]. In agreement with these reports, RT-PCR quantification yielded a copy number for pEH9R in *L. plantarum *WCFS1 of 3 to 4. The ratio of the plasmid copy numbers in the pEH9R- and pEH9A-harboring strains of *L. plantarum *WCFS1 was close to one during the fermentation, meaning that the large differences in β-galactosidase production levels are not due to gene dose effects.

The pSIP409 vector system is based on quorum sensing, because induction by the peptide pheromone also induces transcription of the *sppKR *operon, via the inducible P_sppIP _promoter [6,29,30]. From earlier studies it is known that the transcription levels of the two components of the regulatory system (histidine kinase and response regulator) influence the transcription of the reporter gene [6,29,30]. Studies with reporter genes have shown that the P_sppIP _promoter differs from e.g. the P_sppQ _promoter in that it is more leaky, i.e. it displays more activity under non-inducing conditions [31]. This is supported by our comparative data on the transcription of *sppKR *and *lacLM*, showing that before induction the former operon has higher transcription levels (Table 3). Somewhat surprisingly, transcription of *sppKR *increased only approximately two-fold upon induction, compared to an up to 60-fold increase for the *lacLM *genes controlled by P_sppQ _(in strains harboring pEH9R) (Table 2). The results of previous studies suggest that the *sppKR *transcript is unstable [8]. It is thus conceivable that transcription of these regulatory genes transiently increased to higher levels immediately after addition of the IP, and was already decreasing again two hours later, when the first samples were taken. This may also explain the discrepancy with the results of Risøen et al. [31], who found higher apparent degree of induction using reporter genes. Reporter protein activity can remain stable even after transcription of the encoding gene has ceased and the corresponding mRNA is already degraded and no longer detectable. For the purpose of this study, the most important conclusion is that transcription of *sppKR *in strains harboring either pEH9R or pEH9A is essentially equal, both before and after induction. Variations in the transcription levels of *sppKR *are therefore not responsible for the large differences in the production levels of the two β-galactosidases.

In previous studies of gene regulation in the natural sakacin P producer [8] transcripts for the operon under control of the P_sspQ _promoter could be detected as early as 15 minutes after induction, and maximum levels were reached after 4 hours. Northern blots [8] showed that transcript levels were close to the maximum 2 to 4 hours after induction. In our study, maximum transcript levels for *lacLM *were observed two hours for both pEH9R and pEH9A (Table 2). The mRNA levels were slightly lower 6 hours after induction, i.e. at the start of the stationary phase. The highest activity of β-galactosidase was observed 6 h after induction (12 h of cultivation), indicating an accumulation of the enzyme (Figure 1). After 24 h of cultivation, well into the stationary phase, mRNA of *lacLMReu *was still detected at an 18-fold higher level than before induction (Table 2). In contrast, mRNA levels for *lacLMAci *were lower than before induction at this time point, indicating that *lacLMReu *mRNA is much more stable than *lacLMAci *mRNA in *L. plantarum *WCFS1.

The present data clearly show that the large differences in protein production observed for *lacLMReu *and *lacLMAci *correlate with different mRNA levels. It is unlikely that this is due to differences in the frequency or efficiency of transcription initiation, since the two constructs are identical up to their start codons. Incidental mutations in the two promoter sequences causing different transcriptional efficiency were ruled out by sequencing (data not shown). Thus, translational effects on mRNA production or stability must be the main cause of the large difference in mRNA levels, especially in light of the observed faster decrease in *lacLMAci *mRNA levels, indicating different mRNA stabilities. Translational effects on mRNA levels are often ascribed to the impact of translation on mRNA stability, the main idea being that naked untranslated mRNA is prone to degradation by ribonucleases. It should be noted though that low translation levels will also affect mRNA synthesis directly, either because longer stretches of nascent naked mRNA will be prone to premature Rho-mediated transcription termination [32,33] or because a lack of ribosomes promotes "back-tracking" of the RNA polymerase complex and thus delays transcription, as recently shown by Proshkin et al. [34].

One potential cause of variation in the amount of ribosomes on an emerging mRNA concerns variation in translation initiation frequencies due to variation in the sequence and accessibility of the ribosome-binding site (Shine-Dalgarno-sequence) [35,36]. For example, mRNAs with stable secondary structures near the translational start can hinder ribosome access to the translational initiation region (TIR) (= the ribosome binding site, the start codon and adjacent up- and downstream regions) [37,38]. Analyses using the mfold web server [39] showed only small differences between the two predicted mRNA structures in this region (not shown), but we cannot exclude that these differences play a role.

Another potential cause for slow translation is the presence of rare codons, in particular in the 5' region of the gene [10]. In their recent landmark study on RNA polymerase backtracking [34], Proshkin et al. showed that rare codons not only reduce the speed of translation but also the speed of transcription. Over the entire length of the genes, the two *lacLM *genes used in this study have a similar number of rare codons, but the number of unfavorable codons among the first 50 triplets is considerably higher in *lacLMAci *(seven) than in the better expressed *lacLMReu *(four). As a first step towards investigating the role of rare codons, we replaced two of rare codons the *lacLMAci *by the corresponding less rare codons occurring in the better expressed *lacLMReu *gene. These exchanges included the very rare AGA for arginine in the 15^th ^triplet (frequency 4%) which was replaced by CGC (frequency 21%; note that Arg is a six-fold degenerate amino acid). The mutations indeed yielded an increase in β-galactosidase activity, but the increase was only in the order 25%, and thus far off the approximately 60-fold increase observed when going from *lacLMAci *to *lacLMReu*. While the small increase appears to corroborate our codon-related deliberations in principle, our data seem to indicate that the presence of a few extra rare codons is not sufficient to explain the lower transcription efficiency and/or stability of the *lacLMAci *transcript.

## Conclusion

The results clearly indicate that the much higher β-galactosidase levels obtained in *L. plantarum *harboring *lacLM *from *L. reuteri *(on pEH9R) as compared to *L. plantarum *harboring *lacLM *from *L. acidophilus *(on pEH9A) are caused by higher mRNA levels in the former strain. This is remarkable, since the two operons are expressed using identical transcription and translation machineries and start sequences. This shows the importance of translational effects on mRNA levels. Our data so far indicate that these translational effects are caused by subtle sequence variations at the level of (probably several) rare codons or by minor variations in the secondary structure of the TIR, each of which would affect both mRNA synthesis rates and mRNA stability.

## Competing interests

The authors declare that they have no competing interests.

## Authors' contributions

T-TN, TH-N, TM and GM designed the experiments, T-TN, TM and PS performed the cultivations and quantifications, DH and VGHE conceived of the study, T-TN drafted the manuscript, GM and VGHE contributed to the discussion, CKP supervised research and wrote the final version of the paper. All authors read and approved the final manuscript.
